# The Signature of MicroRNA Dysregulation in Muscle Paralyzed by Spinal Cord Injury Includes Downregulation of MicroRNAs that Target Myostatin Signaling

**DOI:** 10.1371/journal.pone.0166189

**Published:** 2016-12-01

**Authors:** Rita De Gasperi, Zachary A. Graham, Lauren M. Harlow, William A. Bauman, Weiping Qin, Christopher P. Cardozo

**Affiliations:** 1 VA RR&D Service National Center for the Medical Consequences of Spinal Cord Injury, James J. Peters Medical Center, Bronx, New York; 2 Department of Psychiatry, Icahn School of Medicine at Mount Sinai, New York, New York; 3 Department of Medicine, Icahn School of Medicine at Mount Sinai, New York, New York; 4 Department of Rehabilitation Medicine, Icahn School of Medicine at Mount Sinai, New York, New York; 5 Department of Pharmacologic Science Icahn School of Medicine at Mount Sinai, New York, New York; 6 Friedman Brain Institute, Icahn School of Medicine at Mount Sinai, New York, New York; University of Louisville School of Medicine, UNITED STATES

## Abstract

Spinal cord injury (SCI) results in muscle atrophy, reduced force generation and an oxidative-to-glycolytic fiber type shift. The mechanisms responsible for these alterations remain incompletely understood. To gain new insights regarding mechanisms involved in deterioration of muscle after SCI, global expression profiles of miRs in paralyzed gastrocnemius muscle were compared between sham-operated (Sham) and spinal cord-transected (SCI) rats. Ingenuity Pathways Analysis of the altered miRs identified signaling via insulin, IGF-1, integrins and TGF-β as being significantly enriched for target genes. By qPCR, miRs 23a, 23b, 27b, 145, and 206, were downregulated in skeletal muscle 56 days after SCI. Using FISH, miR-145, a miR not previously implicated in the function of skeletal muscle, was found to be localized to skeletal muscle fibers. One predicted target of miR-145 was Cited2, a transcriptional regulator that modulates signaling through NF-κB, Smad3 and other transcription factors. The 3’ UTR of Cited2 mRNA contained a highly conserved miR-145 seed sequence. Luciferase reporter assays confirmed that miR-145 interacts with this seed sequence. However, Cited2 protein levels were similar between Sham and SCI groups, indicating a biochemical interaction that was not involved in the context of adaptations after SCI. Taken together, the findings indicate dysregulation of several highly expressed miRs in skeletal muscle after SCI and suggest that reduced expression of miR-23a, 145 and 206 may have roles in alteration in skeletal muscle mass and insulin responsiveness in muscle paralyzed by upper motor neuron injuries.

## Introduction

Skeletal muscle is a highly plastic tissue that quickly adapts to changes in workload by altering cross sectional area, force production and levels of the metabolic machinery required for oxidative phosphorylation. Immobilization due to bed rest or casting, as well as a wide variety of medical disorders including cancer, infection, renal failure and heart failure, result in muscle atrophy and loss of muscle performance which, ultimately, reduces function and predisposes to frailty. Immobilization or paralysis stimulate rapid decreases in muscle mass and force production, fiber type conversion from slow to mixed or fast-twitch phenotypes, and declines in mitochondrial numbers and capacity for oxidative phosphorylation [1–3]. Key steps in muscle atrophy include catabolism of muscle proteins, particularly myofibrillar proteins, through proteolysis. Most of the protein catabolism that occurs after immobilization or paralysis is mediated through the ubiquitin-proteasome system. Upregulation of at least two muscle-restricted E3 ubiquitin ligases, termed MAFbx and MuRF1, is a common feature of virtually all forms of muscle atrophy studied thus far [1, 4]. Activation of the transcription factors FoxO1 and FoxO3a, NF-κB, and KLF15 are thought to play key roles in the gene expression programs that drive muscle atrophy, including increased expression of MAFbx and MuRF1 [1, 4].

Upstream signals implicated in muscle atrophy caused by immobilization or paralysis include de-novo expression of sarcolemmal connexin 43 and 45 hemichannels [5], activation of NF-κB [6], and signaling via TWEAK/Fn14 [7, 8]. Myostatin may also accelerate the progress of immobilization-related atrophy [9]. Myostatin is a glycoprotein and member of the TGF-β superfamily of growth factors that is released primarily by skeletal and cardiac muscle and is a potent negative regulator of muscle mass [10]. Myostatin signals through binding the Activin receptor IIB, resulting in phosphorylation and nuclear migration of Smad2 and Smad3 and transcriptional regulation of specific target genes [10, 11]. Myostatin signaling is activated in muscle paralyzed by SCI based on findings that mRNA levels for the Activin receptor IIB are elevated and Smad2 and Smad3 are activated in skeletal muscle at 56 days after SCI in rats [12].

A link between microRNAs (miRs) and muscle atrophy has been proposed.[13–15] miRs are small, ~22 base pair non-coding RNAs that are recognized for having major roles in biology and disease.[16–18] miRs bind to sequences in the 3’ UTR of target mRNAs and inhibit translation, promote mRNA degradation or both. In most cases, miRs bind with partial complementarity to one or more sequences within a target mRNA. MyomiRs are miRs that are highly or uniquely expressed in skeletal muscle. The canonical myomirRs are miR-1, miR-133a/b, and miR-206. Other important myomiRs, namely miR-208b, miR-486 and miR-499, have been identified [17]. Dysregulation of miRs has been implicated in muscle atrophy due to nerve transection, spaceflight, bedrest and inflammatory conditions.[13–15, 19, 20]

Spinal cord injury (SCI) results in paresis or paralysis of muscle innervated by motor neurons arising at vertebral levels below the injury site. SCI results in extensive, rapid atrophy of paralyzed muscle with loss of 40–60% of muscle mass, reduced muscle force production, poor endurance and a switch from slow, oxidative to fast glycolytic fiber[2, 3, 21]. Several alterations have been described in muscle obtained from humans with chronic SCI (more than 6 years post injury). As compared to older, sedentary able-bodied male controls, those with SCI showed decreased expression of genes required for glucose storage associated with increased expression of genes for glycolysis [22]. Several alterations have been described in muscle obtained from humans with chronic SCI (more than 6 years post injury). As compared to older, sedentary able-bodied male controls, those with SCI showed decreased expression of genes required for glucose storage associated with increased expression of genes for glycolysis [22]. A comparison of gene expression between able-bodied controls and individuals at 2 and 5 days after SCI found upregulation of ubiquitin ligases (MAFbx, MuRF1), metallothioneins and the proteasome subunit PSMD11 [23]. Shields and co-workers evaluated effects of long-term electrical stimulation (ES) of the soleus muscle in paraplegics using microarray analysis of RNA from soleus muscle biopsies obtained from the FES-trained soleus and its contralateral, untrained counterpart. ES upregulated expression of PGC-1a, slow myosin heavy chain and genes involved in oxidative phosphorylation [24]. Genes that were differentially regulated by starvation, denervation or SCI were interrogated using using connectivity analysis, which identified ursolic acid as a candidate molecule for mitigating these deleterious changes [25]. Follow-up studies in animal models demonstrated that ursolic acid reduced denervation atrophy in mice [25]. The goal of the present study was to further understand the global alterations in miR expression profiles that occur after SCI and to understand the potential relevance of such changes to skeletal muscle atrophy by identifying targets of dysregulated miRs and biological processes to which the target mRNAs contribute. Because of evidence that only the most highly expressed miRs have functional roles [26], our analysis also sought to understand which of the dysregulated miRs are most highly expressed. Our hypothesis was that SCI results in a unique signature of miR dysregulation that predicts alterations in signaling that controls muscle mass, force production and metabolism.

## Materials and Methods

### Animals

Male Wistar-Hannover rats (Taconic Farms) were housed in a temperature and humidity controlled facility with a 12:12 hour light-dark cycle and were provided food and water ad-libitum. SCI involved surgical transection of the spinal cord. Animals were anesthetized by inhalation of isofluorane and weighed. Hair was removed from the skin with a clipper, after which skin was cleaned with an iodine solution and 70% ethanol. The spinal cord was visualized through a midline incision after careful dissection to separate paraspinal muscles from vertebral process and removal of the spinus processes at T4 with a bone rongeur. Several drops of 1% lidocaine were applied to the dura, after which the spinal cord was cut with a fine scissors, and a surgical sponge was placed between the cut ends of the spinal cord. Some animals underwent a sham SCI (Sham). These animals underwent a laminectomy without manipulation of the dura or spinal cord. Animals for the present study were part of a larger study in which Alzet pumps were implanted subcutaneously cephalad to the incision for the SCI or Sham surgery and the wound was closed with suture in layers. The animals described in this study received a continuous infusion of vehicle only (propylene glycol). At 28 days after SCI or sham SCI, animals were anesthetized by inhalation of isofluorane, and pumps were removed and replaced in both groups to continue the vehicle administration.

Post-operatively, animals were administered with carprofen daily for 3 days, Baytril for 5 days, and warmed lactated Ringer’s solution (5 ml) subcutaneously for 3–5 days then as needed. Urine was manually expressed 3 times daily until automaticity of the bladder developed, usually within the first 1–2 weeks, after which urine was expressed as needed. Animals were checked at least daily for the entire course of the study. At 56 days after SCI animals were anesthetized with isofluorane and muscles were excised after isolation by careful dissection, weighed, and snap frozen in liquid nitrogen then stored at -80 until analysis. Normalized muscle weights were calculated by dividing the average weight for the muscle from the left and right sides by body weight prior to surgeries.

Animals were in good health for the duration of the study. One animal in the SCI group died before the 56 day time point due to bladder rupture. All animal studies and procedures were approved by the IACUC at the James J. Peters VA Medical Center and conformed to the NIH Guide for the Care and Use of Laboratory Animals.

### RNA isolation

Total RNA was isolated from gastrocnemius muscle (50–80 mg, random sampling) from the Sham and SCI groups (8 samples/group) using the *mir*Vana miRNA isolation kit (Life Technologies) according to the manufacturer’s instructions. Briefly, the tissue was homogenized in 10 volumes of lysis/binding buffer. The homogenate was extracted with acid-phenol:chloroform and the phases were separated by centrifugation. The aqueous phase was directly loaded onto a glass fiber filter cartridge column to further purify the RNA. Total RNA was eluted with 0.1 mM EDTA (elution solution) preheated at 95°C. The quality of the RNA samples was assessed with an Agilent Bioanalyzer. The average RIN number of the RNA samples was 8.23+/- 0.43.

### Global miRNA expression profiling

miR profiling was performed by Nanostrings Inc using the nCounter rat microRNA assay according to the procedures recommended by the manufacturer. Raw data was processed using nSolver analysis software. Normalization was performed based on the geometric means of the top 100 expressed miRs in each sample. Baseline counts for each sample calculated as the average of the 8 negative controls+2 standard deviations were subtracted from the respective count value. The data were filtered to include the 100 most highly expressed miRs top expressed miR and fold expression change between the Sham and SCI samples was calculated

### Analysis by qPCR of miRNA expression

cDNA was synthesized from 1 ug total RNA using the specific primers provided with the TaqMan miR assays. The RT primers for the miR targets to be quantitated plus the ones for U6 snRNA (normalizer) were pooled, diluted (1:100 final dilution) and the total RNA was reverse transcribed using the TaqMan MicroRNA Reverse transcription kit (Life Technology) according to the protocol provided by the manufacturer (Life Technology protocol #4465407). qPCR was performed using the corresponding TaqMan miR assays in a 10 μl reaction. Normalization was performed using U6 snRNA. The relative expression levels of this snRNA were similar between Sham and SCI samples as indicated by the Ct value reported after analysis of raw qPCR data (21.5+/- 0.17 and 21.12+/- 0.27 respectively). The relative expression of each miR in each sample was calculated with the 2^-ΔΔCt^ method using the Sham samples as reference.[27]

### mRNA target and pathways analysis

To identify known or high probability mRNA targets of the miRs that were differentially expressed in skeletal muscle from SCI rats, we used Ingenuity Pathways Analysis software. The mRNA targets thus identified above were then input into a pathways analysis algorithm in Ingenuity Pathways Analysis (www.ingenuity.com) to identify pathways for which there was a significant overrepresentation of genes.

### Luciferase reporter assays

The 3’UTR of mouse Cited2 (accession NM_010828.3, nucleotides 1051–1853) was amplified from mouse genomic DNA with Platinum Pfx DNA polymerase (Life Technology) and cloned into the pmirGLOW Dual Luciferase vector (Promega) downstream from the luciferase gene. The resulting constructs were sequenced to confirm their identity.

To modify the miR-145 target site in the Cited2-3’UTR, the plasmid was subjected to site directed mutagenesis using the Q5 Site directed mutagenesis kit (New England Biolabs) and the appropriate mutagenic primers (5’AATATGCTAACAGAGAAGATTAAACATGTGGGCCAAAC and 5’TGAAAACTTAAGTCTGTACTC). The miR-145 target site was changed from AACUGGAA to A**CAGA**GAA. The presence of the mutation was verified by sequencing.

Mouse C2C12 cells were grown to 50% confluence in 24 well-plates under proliferating conditions with Dulbecco’s Modified Eagles Medium (Life Technologies) supplemented with 10% fetal bovine serum and 1% penicillin and streptomycin. Cells were co-transfected with 0.2 μg of pmirGLO vector containing the 3’ UTR of Cited2 and a miR-145 mimic (670 nM; Qiagen) or a universal siRNA negative control (Allstars, Qiagen) using the Attractene reagent as per manufacturer’s protocol (Qiagen). The media was replaced 24 h post-transfection. Cells were passively lysed and analyzed 48 h post-transfection for luciferase activity with the Dual-Luciferase Reporter Assay System (Promega) according to the manufacturer’s instructions. Luciferase luminescence values were normalized to *Renilla* luminescence values.

### Fluorescent in situ hybridization (FISH) analysis of miR-145

Frozen rat gastrocnemius muscle sections (8–12 μm) were fixed in ice cold 4% PFA/PBS for 20 min, rinsed twice in RNase-free PBS for 10 min and treated with 20 μg/ml Proteinase K solution (Exiqon) for 5 min. Sections were post-fixed with ice cold 4% PFA/PBS for 20 min, rinsed twice in RNase-free PBS for 5 min and washed in DEPC-treated dH2O. The sections were then rinsed in 0.85% NaCl for 5 min, followed by 2x SSC buffer for 5 min, dehydrated through graded ethanols and left to dry at room temperature for 1 h. The sections were pre-hybridized at 37°C for 4 h in SSC prehybridization solution (bioPlus, 700830) then hybridized overnight at 37°C with a digoxigenin-labeled miRCURY LNA probe for miR-145 or scrambled control (Exiqon) at 20-40nM in hybridization solution (Exiqon). A probe for U6 snRNA (1nM) was used as positive control for the experiment. Following hybridization, the sections were sequentially washed in 2x SSC at 37°C for 15 min, 2x SSC at 50°C for 10 min, 1x SSC for 10 min and 1x SSC/ 0.02% SDS for 10 min. The sections were then washed 4 times for 5 min in PBS/ 0.1% Tween-20 (PBS-T) at room temperature and blocked for 2 h at room temperature in 15% goat serum and 0.1% Triton X-100 in PBS. An anti-digoxigenin HRP Conjugate (PerkinElmer, NEF832001) was applied at 1:200 dilution in blocking solution for 1 h. Sections were then washed with PBS-T 4 times for 5 min each and incubated with 100 μl of Cy5-tyramide working solution (PerkinElmer, NEL745001) for 10 min, washed with PBS-T 4 times for 5 min and air dried. The sections were mounted in DAPI (4',6-diamidino-2-phenylindole)-containing Fluorogel mounting medium (EMS, Hatfield, PA) and imaged with a Zeiss 700 confocal microscope. Images were processed with Adobe Photoshop CC.

### SDS-PAGE and western immunoblotting

A subset of animals (n = 5/group) had cytosolic and nuclear levels of Cited2 protein determined. SDS-PAGE and immunoblotting protocols were similar to previous studies from our lab [12, 28]. Briefly, ~25 mg of gastrocnemius muscle was homogenized and separated into cytosolic and nuclear fractions by a commercially available kit according to the manufacturer’s instructions (NE-PER Nuclear and Cytoplasmic Extraction Kit; Pierce). Protein was quantified by a microBCA kit (Pierce). 60 μg of protein was separated by SDS poly-acrylamide gel and transferred onto a PVDF membrane. After transfer, membranes were blocked with 5% non-fat dry milk in Tris buffered saline, 0.01%Tween (TBST) for 1 h and incubated overnight at 4°C with antibodies against Cited2 (Abcam), ß-tubulin or Histone H3 (Cell Signaling) diluted 1:1000 in TBST /1% non fat dry milk. Membranes were rinsed 3 times for 10 min with TBST and incubated with an anti-rabbit horseradish peroxidase conjugated secondary antibody (Cell Signaling) diluted 1:2000 in TBST 1% milk for 1 h then rinsed 3 times for 10 min. Membranes were then incubated with a chemiluminescent solution (ECL; GE Healthcare) for 5 min and imaged using a CCD digital camera system (AI600, GE Healthcare). Images were quantified using ImageQuant 8.0 (GE Healthcare)

### Data analysis and statistics

Data are expressed as mean valued ± STD. The significance of differences between means was determined with a two-tailed unpaired students *t-test* using Microsoft Excel (Nanostrings data) or Graphpad Prism 7.0. A p value less than 0.05 was considered significant.

## Results

### Changes in body and muscle weight

**Fig 1:**
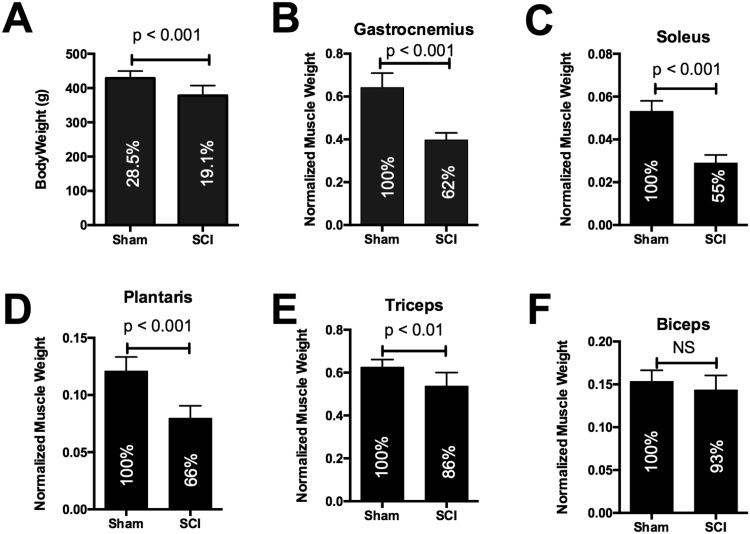
Body and muscle weights at 56 days after spinal cord transection at T4. Data are mean values ± STD for: A) body weight; B) gastrocnemius; C) soleus; D) plantaris; E) triceps; F) biceps. Percentages shown within each bar are: gain in body weight relative to pre-operative weight (panel A) and normalized muscle weight relative to Sham (panels B-F). N = 14 for each group.

Animals in the SCI group had significantly lower body weights at 56 days after SCI (Fig 1A). Weights of gastrocnemius, soleus and plantaris muscles were significantly reduced by SCI (Fig 1B–1D). Weights of triceps muscles, a major postural muscle, were also significantly reduced by SCI, possibly due to reduced workload as a result of diminished mobility and/or body weight (Fig 1E). Biceps weights were not significantly different among groups (Fig 1F).

### Global miR expression profiles

The effect of SCI on global miR expression profiles was examined with an unbiased approach using the Nanostrings miR expression platform. This method determines the number of copies of each of 424 rat miRs. After normalizing raw counts and excluding miRs for which fewer than 100 miR copies were detected, 87 expressed miRs were identified in rat gastrocnemius muscle. Their expression levels ranged from 100 to over 100,000 copies (S1 Table). The number of copies observed for the 20 most-highly expressed miRs varied by over 100-fold (Table 1). The most highly expressed miR was miR-1; its expression was ~4-fold greater than that of miR-206, the next most highly expressed miR (Table 1). The next-most highly expressed miRs were, in descending order, miRs 23a, 378, 22 and 126 (Table 1). miRs 99a, 29a, 210 and let-7a were upregulated whereas miRs 28, 133b, 378, 24, 450a, and 434 were downregulated (Tables 1 and S1).

**Table 1:** Effect of SCI on expression of the mostly highly expressed miRs in gastrocnemius muscle.

miR	MIMAT ID	Mean Sham	Mean SCI	RQ	p value
miR-1	0003125	231068	232084	1.00	0.959
miR-206	0000879	30540	16552	0.54	0.089
miR-133a	0000839	26619	22794	0.86	0.110
miR-29c	0000803	15242	15490	1.02	0.869
miR-378	0003379	14199	11221	0.79	0.019
miR-22	0000791	11521	12154	1.05	0.505
miR-126	0000832	11464	10353	0.90	0.436
miR-16	0000785	10687	11813	1.11	0.081
miR-30c	0000804	10148	10445	1.03	0.719
miR-27b	0000798	8213	7658	0.93	0.172
miR-23a	0000792	8085	7819	0.97	0.680
miR-29a	0000802	6755	8303	1.23	0.038
miR-125b-5p	0000830	6508	7733	1.19	0.105
let-7d	0000562	5473	5348	0.98	0.636
miR-133b	0003126	4960	2640	0.53	0.007
miR-145	0000851	3572	2705	0.78	0.109
miR-30d	0000807	3370	3335	0.99	0.973
miR-199a-3p	0004738	3315	3728	1.12	0.524
let-7i	0000779	3106	3126	1.10	0.897
miR-30a	0000808	2798	2556	0.91	0.497
let-7f	0000778	2691	2708	1.01	0.897
miR-181a	0000858	2249	2418	1.08	0.207
miR-99a	0000820	2209	3859	1.75	0.00001
let-7b	0000775	2121	2042	0.91	0.625
miR-23b	0000793	2090	1898	0.91	0.110

Nanostring analysis was performed on RNA isolated from gastrocnemius from Sham or SCI rats (n = 6 /group). The data were normalized based on the geometric mean of the top 100 expressed miRs in each sample. Expression levels of miRs present at >2000 counts in the Sham group and their expression in the SCI group as compared to the Sham group (RQ) is indicated.

### Networks targeted by differentially expressed miRs

Two hundred and forty nine confirmed targets of the regulated miRs were identified using Ingenuity Pathways Analysis (S2 Table). This list of mRNAs was further analyzed by Ingenuity Pathways Analysis to identify canonical pathways for which there was an over-representation of mRNAs, and to identify upstream regulators of these pathways. Among the top 20 most significant pathways were several related to muscle growth and atrophy including integrin signaling, TGF-ß signaling, ERK/MAPK signaling and role of NFAT in cardiac hypertrophy (Tables 2 and S3). The analysis of upstream mediators revealed TGF-ß1 and TNF-alpha as the second and third most significant categories, respectively (S4 Table).

**Table 2:** Significantly enriched pathways for highly expressed, differentially expressed miRs^1^.

Ingenuity Canonical Pathways	-log(p-value)	Ratio
Axonal Guidance Signaling	4.90E+01	2.26E-01
Molecular Mechanisms of Cancer	3.60E+01	2.11E-01
Protein Kinase A Signaling	3.10E+01	1.90E-01
Integrin Signaling	2.98E+01	2.62E-01
Actin Cytoskeleton Signaling	2.91E+01	2.49E-01
Regulation of the Epithelial-Mesenchymal Transition Pathway	2.89E+01	2.72E-01
Epithelial Adherens Junction Signaling	2.86E+01	3.08E-01
TGF-ß Signaling	2.45E+01	3.79E-01
HGF Signaling	2.37E+01	3.33E-01
B Cell Receptor Signaling	2.29E+01	2.44E-01
Role of Macrophages, Fibroblasts and Endothelial Cells in Rheumatoid Arthritis	2.27E+01	1.85E-01
ERK/MAPK Signaling	2.27E+01	2.35E-01
Glucocorticoid Receptor Signaling	2.23E+01	1.95E-01
Cardiac Hypertrophy Signaling	2.20E+01	2.11E-01
Clathrin-mediated Endocytosis Signaling	2.20E+01	2.32E-01
Role of NFAT in Cardiac Hypertrophy	2.16E+01	2.35E-01
Xenobiotic Metabolism Signaling	2.16E+01	1.89E-01
IGF-1 Signaling	2.15E+01	3.30E-01
Role of Osteoblasts, Osteoclasts and Chondrocytes in Rheumatoid Arthritis	2.15E+01	2.10E-01
Ephrin Receptor Signaling	2.12E+01	2.36E-01
Germ Cell-Sertoli Cell Junction Signaling	2.08E+01	2.44E-01

^1^ Significantly enriched biological themes were determined for the 735 known or predicted mRNA targets for miR-23a, miR-145, miR-206 and miR-467F using Ingenuity Pathways Analysis. The 25 most highly significant pathways identified are shown.

### Quantitation of miR expression by qPCR

**Fig 2:**
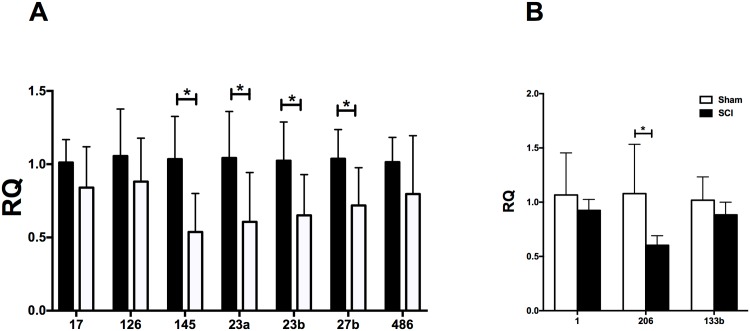
qPCR determinations of relative expression levels of selected miRNAs (panels A and B). Graphs show means ± STD for 8 animals per group. *, p < 0.05. Abbreviations: RQ, fold-change for the comparison between SCI and Sham groups.

Expression of selected miRs was also assessed by qPCR using the same Sham and SCI RNA samples employed for the Nanostrings analysis, as well as two additional samples for each group (total 8/group). miRs were selected for qPCR analysis because they met one of the following criteria: (1) they were myomirs (miRs 1, 133b, and 206) or involved in muscle regeneration (miR-486); (2) they targeted genes implicated in muscle atrophy (miR-23a/b, miR-27); (3) they were expressed in muscle at high levels and were altered after SCI but have unclear roles in muscle atrophy (miR-145); (5) they were not altered after SCI (miRs 17 and 126) and thus provide controls for internal consistency when comparing qPCR and Nanostrings data. As compared to the Sham group, a significant reduction in miR expression was observed in the SCI group for miR-23a, miR-23b, miR-27b and, miR-145 (Fig 2A). Expression of miRs 17, 126, and 486 was not different between Sham and SCI groups (Fig 2A). Among the myomiRs, miR-206 was significantly downregulated after SCI whereas no change in expression was observed for miR-1 or miR-133b (Fig 2B).

Comparison of the qPCR and Nanostrings data revealed agreement for miR-1, miR-126, and miR-17, which were unchanged after SCI by both methods (Fig 2, Tables 1 and S1). By qPCR, miR-145 and miR-206 were downregulated after SCI (Fig 2); their expression was reduced after SCI by Nanostrings although the differences were not significant (p < 0.11 and 0.08, respectively) (Fig 2 and Table 1). miR-23a and b were found to be reduced by qPCR (Fig 2) and were reduced by Nanostrings although not significantly so (p < 0.68 and p < 0.11, respectively) (Tables 1 and S1). miR-133b was reduced in the Nanostrings analysis but unchanged by qPCR.

### Distribution of miR-145 in muscle tissue

**Fig 3:**
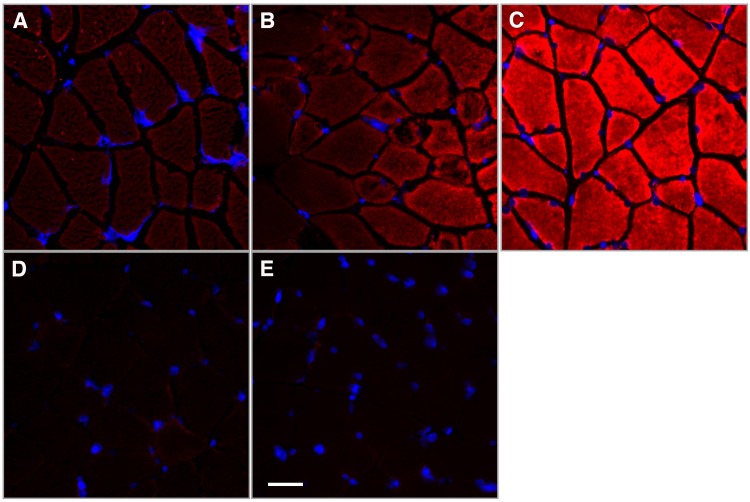
miR-145 is localized to muscle fibers. LNA-FISH was performed on cross sections of (rat gastrocnemius muscle using DIG-labeled LNA-FISH probes for miR-145, scrambled control, and U6 snRNA. A: 20 nM miR-145 probe, B: 40 nM miR-145 probe; C: U6 snRNA probe (1 nM); D: scrambled control probe (40 nM); E: no probe. Scale bar: 30 microns.

As noted above, one of the miRs downregulated after SCI was miR-145, which has been reported to be present in skeletal muscle homogenates and to undergo reduced expression after denervation or starvation [15]. It is not known whether miR-145 is present in skeletal muscle fibers as opposed to other cellular constituents of skeletal muscle, and consequently or whether such reductions might impact skeletal muscle fiber homeostasis. Therefore, distribution within muscle of miR-145 was addressed using LNA-FISH. Cross sections of rat gastrocnemius muscle revealed bright staining for miR-145 within muscle fibers in proportion to the concentration of the hybridization probe (Fig 3A and 3B) while no staining was observed with a scrambled miR control (Fig 3D) or without any probe (Fig 3E) confirming that miR-145 is expressed in skeletal muscle fibers. Intense staining was noted with a probe for U6 snRNA used as positive control for the experiment (Fig 3C).

### miRs altered after SCI by target multiple mRNA in TGF-ß signaling

The identification of TGF-ß signaling as a pathway for which there was an over-representation of confirmed mRNA targets of miRs that were differentially regulated in the above Nanostrings raised the question as to the identity of the targets of the dysregulated miRs that are involved in TGF-ß signaling. This question is important given that signaling downstream of myostatin is activated after SCI [12] and that myostatin is a potent negative regulator of muscle size [29, 30]. To address this question, an additional bioinformatics analysis was performed to identify confirmed and predicted mRNA targets of the miRs that were downregulated after SCI by qPCR and are involved in TGF-ß signaling. mRNA targets were identified using Ingenuity Pathways Analysis and MirWalk (www.mirwalk.com). Predicted targets were included in this analysis in order to test the possibility that potential targets of the dysregulated miRs included mRNA transcripts that had not been previously validated.

Confirmed or predicted mRNA targets involved in TGF-β signaling were identified for miR-23a, miR-145, and miR-206. The targets included receptors (AcvR1B, AcvR1C, AcvR2A, AcvR2B), transcription factors (Smad2, Smad3, Smad4, Smad5) and the transcriptional co-regulator Cited2 (Table 3). Six mRNA targets involved in TGF-β signaling were identified for miR-23a, 9 for miR-145, and 5 for miR-206 (Table 3). Three confirmed targets (AcvR-IB, Smad3 and Smad4) were identified (Table 3).

**Table 3:** mRNAs targets of differentially expressed miRs that lie within the signaling pathways of TGFß family members. Confirmed targets are identifed by boldface. Numbers in brackets are literature citations for confirmed targets.

miR	mRNA
miR-23a	AcvR-IC
	AcvR-IIB
	BMRP1B
	BMPR2
	Smad3
	**Smad4** [63]
	Smad5
miR-145	**AcvR-IB** [47, 48]
	AcvR-IIA
	Cited2
	Inhibin
	Smad2
	**Smad3** [47, 48]
	Smad4
	Smad5
	TGFBR2
	AcvR-IIB
miR-206	BMPR-1B
	Smad2
	Smad4
	TGFBR3
	Cited2

### Cited2 mRNA is a miR-145 target

**Fig 4:**
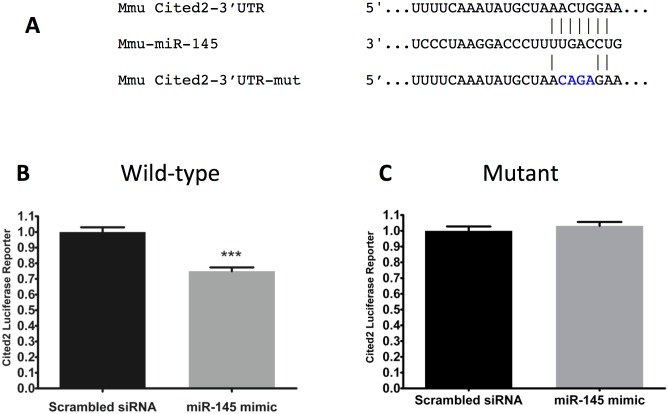
miR-145 targets Cited2. A. Alignment of miR-145 with the putative target site in the 3’UTR of Cited2 and with a mutated version. B. Luciferase reporter gene assays were conducted after transfection of C2C12 cells with reporter constructs harboring the Cited2 3’UTR containing a wild-type or mutant miR-145 seed sequence and with a miR-145 mimic or a scrambled RNA control. Data are the combined mean values ± STD of two separate experiments; n = 12 wells.

The possibility that Cited2 is indeed a target for miR-145 was considered. Analysis using the TargetScan miR prediction program (http://www.targetscan.org) revealed a miR-145 seed sequence in the 3’-UTR of the mouse Cited2 mRNA. An alignment of the 3’ UTR of Cited2 from 23 species was performed and revealed that the miR-145 seed sequence found in the mouse Cited2 mRNA is highly conserved across 20 species, including rats (S1 Fig) but was absent from guinea pig (Cpo) and cat (Fca), and mutated in frog (Xtr). Reporter gene studies were performed to test whether miR-145 altered the expression of a luciferase reporter into which a portion the 3’ UTR of the Cited2 mRNA containing the miR-145 seed sequence or a mutated version of it was inserted 3’ to the coding sequence for the firefly luciferase gene. miR-145 significantly reduced normalized firefly luciferase activity whereas mutation of the predicted miR-145 seed sequence in the 3’ UTR of the Cited2 mRNA abrogated this effect (Fig 4A and 4B).

### Cited2 protein levels after SCI

**Fig 5:**
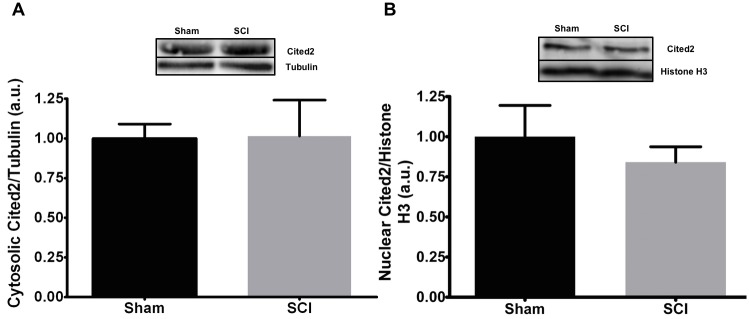
Cited2 protein expression is maintained at 56 days post-SCI. Rat gastrocnemius muscle was fractionated into cytosolic (A) and nuclear fractions (B) and probed for Cited2 expression by immunoblotting. There were no differences in either the cytosolic or nuclear expression of Cited2. Data are presented as mean values normalized to tubulin or histone H3. N = 5 per group.

To determine miR-145 was correlated with in expression of Cited2, rat gastrocnemius muscle was subjected to subcellular fractionation. Cytosolic and nuclear Cited2 levels were determined in lysates of gastrocnemius muscles. Neither cytosolic nor nuclear Cited2 protein levels were significantly different between Sham and SCI groups (Fig 5A and 5B, respectively).

## Discussion

Studies using microarrays have identified approximately 150 miRs in skeletal muscle [15]. Which of these participate in homeostasis or responses to changes in workload, injury or disease is not yet fully understood. Knowledge regarding the relative abundance of these miRs is important to understanding which miRs contribute to muscle homeostasis because it was recently reported that only the most abundant miRs have discernable functions based upon screens with sensing and inhibitory libraries [26]. The Nanostrings platform employed for profiling miR expression measures the number of copies of each miR species present in each sample. This permitted the identification of the most highly expressed miRs present in gastrocnemius muscle from male rats. By far the most abundant miR was miR-1. If one takes an abundance of 10% of that for miR-1 as a cut-off for being highly expressed, only miR-206 and miR 29c would be included. Using a cutoff of 1% of the abundance of that of miR-1, a total of 22 miRs would be present at concentrations above the cutoff; these miRs include miRs 23a, and 145.

Parallel studies were performed using qPCR to further understand how SCI altered expression levels of specific miRs. This second approach was intended to evaluate changes in the expression of specific miRs such as the myomirs, mirRs implicated either directly (miR-206) or indirectly (miR-23a/b) in muscle atrophy, and one miR not previously linked to muscle atrophy (miR-145). Several additional miRs that were not altered by Nanostrings analysis were included to validate any comparisons between results of the two assays. All samples used in the Nanostrings comparison were also analyzed by qPCR assuring that the two analyses were comparable.

In general, the direction of changes observed were in agreement between the pPCR and Nanostrings data with the caveat that difference did not reach the threshold for significance using the latter approach in all cases. There may be several explanations for these differences. The most obvious is that more samples were analyzed by qPCR. In addition, qPCR data were normalized relative to expression of a housekeeping gene, U6 RNA whereas Nanostrings data were normalized using on an adjustment to absolute counts based on the geometric mean for the top 100 miRs, some of which were below the cutoff for inclusion in our analysis. In addition, the signal to noise ratio for miRs detected at less than 1000 copies per samples is, presumably, low relative to that for the qPCR approach resulting in greater variability with the Nanostrings method. The qPCR data support the conclusion that 5 highly expressed miRs are downregulated after SCI: miRs 23a, 23b, 27b, 145 and 206. The Nanostrings data are consistent with this conclusion although reductions in levels of these miRs by Nanostrings that did not reach significance. We therefore conclude that at 56 days after a spinal cord transection there is reduced expression of miRs 23a, 23b, 27b, 145 and 206. Due to the heterogeneity of fiber type in the gastrocnemius, some variability may have been introduced by the random sampling of the tissue for RNA isolation, however this did not obscure any significant differences in the expression of the above miRs. A consideration is that the surgery to replace the mini-osmotic pumps may have resulted in a stress that affected skeletal muscle homeostasis and gene regulation. It should be noted that both Sham and SCI groups underwent this procedure which we believe would provide a control for any potential confounding effects of this surgery on miR expression. While effects of these surgeries on muscle were not examined in the current study, we have previously reported that expression of several muscle atrophy genes is increased at early time points after either laminectomy [31], and that these alterations resolve by 14 days. While we cannot exclude an influence of these surgeries on skeletal muscle in the Sham and SCI rats used in the current study, our prior results suggest that any effects are small relative to those resulting from the SCI itself.

The alterations in miR expression following SCI resemble, in part, those observed after bedrest or space flight. Following 10 days of bedrest, 15 miRs were downregulated in biopsies from the vastus lateralis, including miR-23a and miR-206, and let-7 family members [13]. After 11 days and 19 hours in space, rat gastrocnemius muscle revealed reductions in miR-206 and trends toward decreases in miR-1 [14]. When taken together, these findings suggest that downregulation of miR-206 may be regarded as a characteristic of immobilized muscle. A similar pattern of dysregulation of miR-1, 133a, 133b and 206 has been reported in inflammatory myopathies and in a cell culture system in which TNF-α reduced expression of these myomiRs [20]. It should be noted, however, that the changes in miR expression observed after upper motor neuron injury caused by SCI or from spaceflight are strikingly different from those reported after severing lower motor neurons by nerve transection. As an example, miR-21 and miR-206 are upregulated early after nerve transection [15].

Muscle atrophy after SCI develops rapidly. In rats, 40–60% of muscle mass is lost within the first 2 weeks after SCI and muscle mass appears to decrease slowly thereafter, if at all [12, 32, 33]. Key genes involved in the early stages of muscle atrophy after SCI are likely to include MAFbx and MuRF1, two E3 ubiquitin ligases that have been shown to accelerate atrophy in several animal models [4, 34, 35]. In animal models, expression of these transcripts is increased over the first one to two weeks after SCI, but returns to baseline levels at later time points [12, 32, 33]. There is evidence suggesting that reduced expression of miR-23a may exacerbate muscle atrophy. Using computer-based predictions miR-23a and miR-23b were identified as the only miRs that target both MAFbx and MuRF1 [4] and reporter gene studies confirmed MAFbx and MuRF1 as targets [36]. Overexpression of miR-23a protected C2C12 myotubes against glucocorticoid-induced atrophy and miR-23a transgenic mice were resistant to GC-induced skeletal muscle atrophy [36] by inhibiting signaling through TGF-ß/Smad2/Smad3.

Several studies indicate that miR-206 plays a role in regeneration of synapses and in protecting muscle from atrophy through the downregulation of HDAC4 [37–39]. miR-206 was found to be upregulated in muscle after nerve transection [15] or after nerve crush [40]. There is conflicting evidence as to the role of miR-206 in denervation atrophy. In one report using overexpression or knockdown approaches, miR-206 was observed to stimulate denervation atrophy [15]. In contrast, injection of miR-206 in rat gastrocnemius muscle following nerve transection attenuated atrophy via inhibition of TGF-β/Smad3 signaling [38]. Thus, the implications to muscle atrophy of the reduced levels of miR-206 observed in muscle at 56 days after SCI remain uncertain.

miR-145 was identified as a being downregulated in skeletal muscle at 56 days after SCI. Consistent with our findings, a global screen of miR expression changes under different muscle wasting conditions found that miR-145 was downregulated after denervation or starvation [15]. Its function in skeletal muscle is uncertain. miR-145 has been found to have roles in the pathogenesis of many different types of cancer and to be an important modulator of smooth muscle [41], in part through modulation of KLF4 [42]. In cardiomyocytes, miR-145 has been shown to prevent damage induced by reactive oxygen species through protection against mitochondrial apoptosis [43], to protect against Ca^2+^ overload induced by reactive oxygen species by targeting CaMKIIδ [44] and to block cardiomyocyte hypertrophy by targeting GATA6 [45]. Its ability to target Activin receptor IB [46] and Smad3 [47, 48] suggest that reduced levels of miR-145 would promote muscle atrophy gene expression programs.

One objective of our study was to identify mRNA targets of dysregulated miRs that might contribute to muscle atrophy but had not yet been validated. The sequence alignment and reporter gene studies support the conclusion that miR-145 binds the predicted seed sequence within the Cited2 3’ UTR and represses translation of Cited2 protein. The physiologic importance of this activity of miR-145 in skeletal muscle is, however, unclear as nuclear and cytoplasmic Cited2 protein levels were not altered in muscle from SCI rats despite the reduction of miR-145 observed after SCI. Possible explanations for these discrepant findings are that miR-145 levels in muscle fibers are too low to influence Cited2 translation or that Cited2 protein levels are controlled by protein turnover.

The findings confirm that Cited2 protein is expressed in skeletal muscle, presumably within skeletal muscle as well as other cellular elements of muscle. The role(s) of Cited2 in skeletal muscle have not been elucidated. Cited2 is a transcriptional co-regulator that, through binding to p300, modulates the activity of many transcription factors including HIF1 [49], Ets-1 [50], PPARα [51] and HNF4α [52]. In C2C12 cells, Cited2 overexpression resulted in myotube sparing effects in the presence of dexamethasone through its interaction with p300/CBP and possible inhibition of NF-κB activation, a critical driver of skeletal muscle atrophy [6], suggesting a protective role of Cited2 against the catabolic effects of glucocorticoid [53]. In fact, Cited2 has been shown to reduce binding of NF-κB to its cognate promoter binding sites [54] and to sensitize cells to TNFα-induced apoptosis. Cited2 is necessary for cardiac [55], hepatic [52] and pulmonary [56], proper regulation of energy metabolism[57, 58] and cell fate decisions [59]. In addition, Cited2 was reported to increase TGF-β induced expression of MMP9 while a knockdown of Cited2 blunted this response; in these studies Cited2 and Smad3 were recruited to the MMP9 promoter by TGF-β treatment, suggesting direct effects of Cited2 on transcriptional activity of the Smad2/3/4 complex [60]. Therefore, in skeletal muscle Cited2 may also modulate signal transduction by Smad2, Smad3 and Smad4 downstream of myostatin. Cited2 has also been shown to play critical roles in responses of tissues of mesenchymal origin such as fracture healing and responses of cartilage to loading [61, 62].

In summary, we found by qPCR that 5 highly expressed miRs are downregulated by the upper motor neuron lesion caused by SCI. The down-regulated miRs target key signaling pathways involved in muscle atrophy, insulin action and the insulin and IGF-1 pathways, and TGF-ß signaling. Down-regulated miRs also suppress expression of key atrophy genes, the E3 ubiquitin ligases MAFbx and MuRF1. Downregulation of these miRs after SCI may contribute to the chronic reduction in muscle size and insulin sensitivity of skeletal muscle.

## Supporting Information

S1 Fig A conserved, predicted miR-145 seed sequence was identified in the 3’-UTR of mouse Cited2 mRNA. Upper panel shows a sequence alignment of the region of mouse (Mmu) Cited2 mRNA containing the predicted miR-145 seed sequence; the seed sequence is highlighted in light blue. Lower panel: an alignment of murine cited2 mRNA and murine miR-145 is shown. The seed sequence within the Cited2 3’-UTR is highlighted in red.
(TIFF)

S1 Table 87 miRs were identified in rat gastrocnemius muscle. Three worksheets are included. (1) “NS_R_MIR_raw data”: these are the raw data obtained by the chip reader; (2) “top-100 miR normalized data”: these are the normalized data; (3) “Selected Data”: these are data that met filtering criteria after normalization.
(XLSX)

S2 Table mRNA targets for differentially regulated miRs. Validated targets for differentially regulated miRs were identified by Ingenuity Pathways Analysis. Results of the analysis were exported as a text file which was then imported into Microsoft Excel.
(XLSX)

S3 Table Pathways represented by mRNA targets for differentially regulated miRs. Ingenuity Pathways Analysis was used to identify pathways represented by mRNA targets of differentially regulated miRs. Results of the analysis were exported as a text file which was then imported into Microsoft Excel.
(XLS)

S4 Table Upstream mediators that regulate the mRNA targets in S3 Table including miRs. Ingenuity Pathways analysis was used to identify the upstream mediators that regulate expression of the mRNAs listed in S3 Table that were mRNA targets for differentially regulated miRs. Results of the analysis were exported as a text file which was then imported into Microsoft Excel.
(XLS)

## References

[1] Bodine SC. Disuse-induced muscle wasting. Int J Biochem Cell Biol. 2013;45(10):2200–8. Epub 2013/06/27. 10.1016/j.biocel.2013.06.011 23800384 PMC3856924

[2] Dudley-Javoroski S, Shields RK. Muscle and bone plasticity after spinal cord injury: review of adaptations to disuse and to electrical muscle stimulation. J Rehabil Res Dev. 2008;45(2):283–96. Epub 2008/06/21. 18566946 10.1682/jrrd.2007.02.0031 PMC2744487

[3] Qin W, Bauman WA, Cardozo C. Bone and muscle loss after spinal cord injury: organ interactions. Ann N Y Acad Sci. 2010;1211(1):66–84. Epub 2010/11/11. 21062296 10.1111/j.1749-6632.2010.05806.x

[4] Bodine SC, Baehr LM. Skeletal muscle atrophy and the E3 ubiquitin ligases MuRF1 and MAFbx/atrogin-1. Am J Physiol Endocrinol Metab. 2014;307(6):E469–E84. Epub 2014/08/07. 10.1152/ajpendo.00204.2014 25096180 PMC4166716

[5] Cea LA, Cisterna BA, Puebla C, Frank M, Figueroa XF, Cardozo C, et al De novo expression of connexin hemichannels in denervated fast skeletal muscles leads to atrophy. Proc Natl Acad Sci U S A. 2013;110(40):16229–34. Epub 2013/09/18. 10.1073/pnas.1312331110 24043768 PMC3791696

[6] Jackman RW, Cornwell EW, Wu CL, Kandarian SC. Nuclear factor-kappaB signalling and transcriptional regulation in skeletal muscle atrophy. Exp Physiol. 2013;98(1):19–24. Epub 2012/08/01. 10.1113/expphysiol.2011.063321 22848079 PMC3505235

[7] Sato S, Ogura Y, Kumar A. TWEAK/Fn14 Signaling Axis Mediates Skeletal Muscle Atrophy and Metabolic Dysfunction. Frontiers in immunology. 2014;5:18 Epub 2014/01/31. 10.3389/fimmu.2014.00018 24478779 PMC3902304

[8] Mittal A, Bhatnagar S, Kumar A, Lach-Trifilieff E, Wauters S, Li H, et al The TWEAK-Fn14 system is a critical regulator of denervation-induced skeletal muscle atrophy in mice. J Cell Biol. 2010;188(6):833–49. Epub 2010/03/24. 10.1083/jcb.200909117 20308426 PMC2845082

[9] Murphy KT, Cobani V, Ryall JG, Ibebunjo C, Lynch GS. Acute antibody-directed myostatin inhibition attenuates disuse muscle atrophy and weakness in mice. J Appl Physiol. 2011;110(4):1065–72. Epub 2011/01/29. 10.1152/japplphysiol.01183.2010 21270350

[10] Lee SJ. Regulation of muscle mass by myostatin. Annu Rev Cell Dev Biol. 2004;20:61–86. 10.1146/annurev.cellbio.20.012103.135836 15473835

[11] Glass DJ. Skeletal muscle hypertrophy and atrophy signaling pathways. Int J Biochem Cell Biol. 2005;37(10):1974–84. 10.1016/j.biocel.2005.04.018 16087388

[12] Wu Y, Zhao J, Zhao W, Pan J, Bauman WA, Cardozo CP. Nandrolone normalizes determinants of muscle mass and fiber type after spinal cord injury. J Neurotrauma. 2012;29(8):1663–75. Epub 2012/01/03. 10.1089/neu.2011.2203 22208735 PMC5364642

[13] Rezen T, Kovanda A, Eiken O, Mekjavic IB, Rogelj B. Expression changes in human skeletal muscle miRNAs following 10 days of bed rest in young healthy males. Acta Physiol (Oxf). 2014;210(3):655–66. 24410893 10.1111/apha.12228

[14] Allen DL, Bandstra ER, Harrison BC, Thorng S, Stodieck LS, Kostenuik PJ, et al Effects of spaceflight on murine skeletal muscle gene expression. J Appl Physiol (1985). 2009;106(2):582–95. 19074574 10.1152/japplphysiol.90780.2008 PMC2644242

[15] Soares RJ, Cagnin S, Chemello F, Silvestrin M, Musaro A, De Pitta C, et al Involvement of microRNAs in the regulation of muscle wasting during catabolic conditions. J Biol Chem. 2014;289(32):21909–25. Epub 2014/06/04. 10.1074/jbc.M114.561845 24891504 PMC4139209

[16] Kirby TJ, Chaillou T, McCarthy JJ. The role of microRNAs in skeletal muscle health and disease. Front Biosci (Landmark Ed). 2015;20:37–77. 25553440 10.2741/4298 PMC4853752

[17] Hitachi K, Tsuchida K. Role of microRNAs in skeletal muscle hypertrophy. Front Physiol. 2013;4:408 10.3389/fphys.2013.00408 24474938 PMC3893574

[18] McCarthy JJ. MicroRNA-206: the skeletal muscle-specific myomiR. Biochim Biophys Acta. 2008;1779(11):682–91. 10.1016/j.bbagrm.2008.03.001 18381085 PMC2656394

[19] McCarthy JJ, Esser KA, Peterson CA, Dupont-Versteegden EE. Evidence of MyomiR network regulation of beta-myosin heavy chain gene expression during skeletal muscle atrophy. Physiol Genomics. 2009;39(3):219–26. 10.1152/physiolgenomics.00042.2009 19690046 PMC2789671

[20] Georgantas RW, Streicher K, Greenberg SA, Greenlees LM, Zhu W, Brohawn PZ, et al Inhibition of myogenic microRNAs 1, 133, and 206 by inflammatory cytokines links inflammation and muscle degeneration in adult inflammatory myopathies. Arthritis Rheumatol. 2014;66(4):1022–33. 10.1002/art.38292 24757153

[21] Biering-Sorensen B, Kristensen IB, Kjaer M, Biering-Sorensen F. Muscle after spinal cord injury. Muscle Nerve. 2009;40(4):499–519. Epub 2009/08/26. 10.1002/mus.21391 19705475

[22] Long YC, Kostovski E, Boon H, Hjeltnes N, Krook A, Widegren U. Differential expression of metabolic genes essential for glucose and lipid metabolism in skeletal muscle from spinal cord injured subjects. J Appl Physiol. 2011;110(5):1204–10. Epub 2011/03/12. 10.1152/japplphysiol.00686.2010 21393466

[23] Urso ML, Chen YW, Scrimgeour AG, Lee PC, Lee KF, Clarkson PM. Alterations in mRNA expression and protein products following spinal cord injury in humans. J Physiol. 2007;579(Pt 3):877–92. Epub 2007/01/16. 10.1113/jphysiol.2006.118042 17218363 PMC2151363

[24] Adams CM, Suneja M, Dudley-Javoroski S, Shields RK. Altered mRNA expression after long-term soleus electrical stimulation training in humans with paralysis. Muscle Nerve. 2011;43(1):65–75. Epub 2010/12/21. 10.1002/mus.21831 21171097 PMC3058836

[25] Kunkel SD, Suneja M, Ebert SM, Bongers KS, Fox DK, Malmberg SE, et al mRNA expression signatures of human skeletal muscle atrophy identify a natural compound that increases muscle mass. Cell Metab. 2011;13(6):627–38. 10.1016/j.cmet.2011.03.020 21641545 PMC3120768

[26] Mullokandov G, Baccarini A, Ruzo A, Jayaprakash AD, Tung N, Israelow B, et al High-throughput assessment of microRNA activity and function using microRNA sensor and decoy libraries. Nature methods. 2012;9(8):840–6. Epub 2012/07/04. 10.1038/nmeth.2078 22751203 PMC3518396

[27] Livak KJ, Schmittgen TD. Analysis of relative gene expression data using real-time quantitative PCR and the 2(-Delta Delta C(T)) method. Methods. 2001;25(4):402–8. 10.1006/meth.2001.1262 11846609

[28] Graham ZA, Qin W, Harlow LC, Ross NH, Bauman WA, Gallagher PM, et al Focal adhesion kinase signaling is decreased 56 days following spinal cord injury in rat gastrocnemius. Spinal Cord. 2015. 10.1038/sc.2015.183 26481700

[29] Ruegg MA, Glass DJ. Molecular mechanisms and treatment options for muscle wasting diseases. Annu Rev Pharmacol Toxicol. 2011;51:373–95. Epub 2010/10/13. 10.1146/annurev-pharmtox-010510-100537 20936944

[30] Glass DJ. Signaling pathways perturbing muscle mass. Curr Opin Clin Nutr Metab Care. 2010;13(3):225–9. Epub 2010/04/20. 20397318 10.1097/mco.0b013e32833862df

[31] Wu Y, Hou J, Collier L, Pan J, Hou L, Qin W, et al The administration of high-dose methylprednisolone for 24 h reduced muscle size and increased atrophy-related gene expression in spinal cord-injured rats. Spinal Cord. 2011;49(8):867–73. Epub 2011/03/30. 10.1038/sc.2011.28 21445080

[32] Zeman RJ, Zhao J, Zhang Y, Zhao W, Wen X, Wu Y, et al Differential skeletal muscle gene expression after upper or lower motor neuron transection. Pflugers Arch. 2009;458(3):525–35. Epub 2009/02/14. 10.1007/s00424-009-0643-5 19214561

[33] Wu Y, Collier L, Qin W, Creasey G, Bauman WA, Jarvis J, et al Electrical stimulation modulates Wnt signaling and regulates genes for the motor endplate and calcium binding in muscle of rats with spinal cord transection. BMC neuroscience. 2013;14(1):81. Epub 2013/08/07. 23914941 10.1186/1471-2202-14-81 PMC3735397

[34] Waddell DS, Baehr LM, van den Brandt J, Johnsen SA, Reichardt HM, Furlow JD, et al The Glucocorticoid Receptor and Foxo1 Synergistically Activate the Skeletal Muscle Atrophy Associated Murf1 Gene. Am J Physiol Endocrinol Metab. 2008;295(4):E785–97. 10.1152/ajpendo.00646.2007 18612045 PMC2652500

[35] Bodine SC, Latres E, Baumhueter S, Lai VK, Nunez L, Clarke BA, et al Identification of ubiquitin ligases required for skeletal muscle atrophy. Science. 2001;294(5547):1704–8. 10.1126/science.1065874 11679633

[36] Wada S, Kato Y, Okutsu M, Miyaki S, Suzuki K, Yan Z, et al Translational suppression of atrophic regulators by microRNA-23a integrates resistance to skeletal muscle atrophy. J Biol Chem. 2011;286(44):38456–65. Epub 2011/09/20. 10.1074/jbc.M111.271270 21926429 PMC3207415

[37] Liu N, Williams AH, Maxeiner JM, Bezprozvannaya S, Shelton JM, Richardson JA, et al microRNA-206 promotes skeletal muscle regeneration and delays progression of Duchenne muscular dystrophy in mice. J Clin Invest. 2012;122(6):2054–65. 10.1172/JCI62656 22546853 PMC3366415

[38] Huang QK, Qiao HY, Fu MH, Li G, Li WB, Chen Z, et al MiR-206 Attenuates Denervation-Induced Skeletal Muscle Atrophy in Rats Through Regulation of Satellite Cell Differentiation via TGF-beta1, Smad3, and HDAC4 Signaling. Med Sci Monit. 2016;22:1161–70. 10.12659/MSM.897909 27054781 PMC4829125

[39] Williams AH, Valdez G, Moresi V, Qi X, McAnally J, Elliott JL, et al MicroRNA-206 delays ALS progression and promotes regeneration of neuromuscular synapses in mice. Science. 2009;326(5959):1549–54. 10.1126/science.1181046 20007902 PMC2796560

[40] Wiberg R, Jonsson S, Novikova LN, Kingham PJ. Investigation of the Expression of Myogenic Transcription Factors, microRNAs and Muscle-Specific E3 Ubiquitin Ligases in the Medial Gastrocnemius and Soleus Muscles following Peripheral Nerve Injury. PLoS One. 2015;10(12):e0142699 10.1371/journal.pone.0142699 26691660 PMC4686181

[41] Cordes KR, Sheehy NT, White MP, Berry EC, Morton SU, Muth AN, et al miR-145 and miR-143 regulate smooth muscle cell fate and plasticity. Nature. 2009;460(7256):705–10. 10.1038/nature08195 19578358 PMC2769203

[42] Davis-Dusenbery BN, Chan MC, Reno KE, Weisman AS, Layne MD, Lagna G, et al down-regulation of Kruppel-like factor-4 (KLF4) by microRNA-143/145 is critical for modulation of vascular smooth muscle cell phenotype by transforming growth factor-beta and bone morphogenetic protein 4. J Biol Chem. 2011;286(32):28097–110. 10.1074/jbc.M111.236950 21673106 PMC3151055

[43] Li R, Yan G, Li Q, Sun H, Hu Y, Sun J, et al MicroRNA-145 protects cardiomyocytes against hydrogen peroxide (H(2)O(2))-induced apoptosis through targeting the mitochondria apoptotic pathway. PLoS One. 2012;7(9):e44907 10.1371/journal.pone.0044907 23028672 PMC3445575

[44] Cha MJ, Jang JK, Ham O, Song BW, Lee SY, Lee CY, et al MicroRNA-145 suppresses ROS-induced Ca2+ overload of cardiomyocytes by targeting CaMKIIdelta. Biochem Biophys Res Commun. 2013;435(4):720–6. 10.1016/j.bbrc.2013.05.050 23702479

[45] Li R, Yan G, Zhang Q, Jiang Y, Sun H, Hu Y, et al miR-145 inhibits isoproterenol-induced cardiomyocyte hypertrophy by targeting the expression and localization of GATA6. FEBS Lett. 2013;587(12):1754–61. 10.1016/j.febslet.2013.04.018 23624080 PMC4183134

[46] Yan G, Zhang L, Fang T, Zhang Q, Wu S, Jiang Y, et al MicroRNA-145 suppresses mouse granulosa cell proliferation by targeting activin receptor IB. FEBS Lett. 2012;586(19):3263–70. 10.1016/j.febslet.2012.06.048 22796494

[47] Huang H, Sun P, Lei Z, Li M, Wang Y, Zhang HT, et al miR-145 inhibits invasion and metastasis by directly targeting Smad3 in nasopharyngeal cancer. Tumour Biol. 2015;36(6):4123–31. 10.1007/s13277-015-3046-6 25578496

[48] Megiorni F, Cialfi S, Cimino G, De Biase RV, Dominici C, Quattrucci S, et al Elevated levels of miR-145 correlate with SMAD3 down-regulation in cystic fibrosis patients. J Cyst Fibros. 2013;12(6):797–802. 10.1016/j.jcf.2013.03.007 23632450

[49] Freedman SJ, Sun ZY, Kung AL, France DS, Wagner G, Eck MJ. Structural basis for negative regulation of hypoxia-inducible factor-1alpha by CITED2. Nature structural biology. 2003;10(7):504–12. 10.1038/nsb936 12778114

[50] Yokota H, Goldring MB, Sun HB. CITED2-mediated regulation of MMP-1 and MMP-13 in human chondrocytes under flow shear. J Biol Chem. 2003;278(47):47275–80. 10.1074/jbc.M304652200 12960175

[51] Tien ES, Davis JW, Vanden Heuvel JP. Identification of the CREB-binding protein/p300-interacting protein CITED2 as a peroxisome proliferator-activated receptor alpha coregulator. J Biol Chem. 2004;279(23):24053–63. 10.1074/jbc.M401489200 15051727

[52] Qu X, Lam E, Doughman YQ, Chen Y, Chou YT, Lam M, et al Cited2, a coactivator of HNF4alpha, is essential for liver development. EMBO J. 2007;26(21):4445–56. 10.1038/sj.emboj.7601883 17932483 PMC2063472

[53] Tobimatsu K, Noguchi T, Hosooka T, Sakai M, Inagaki K, Matsuki Y, et al Overexpression of the transcriptional coregulator Cited2 protects against glucocorticoid-induced atrophy of C2C12 myotubes. Biochem Biophys Res Commun. 2009;378(3):399–403. 10.1016/j.bbrc.2008.11.062 19032942

[54] Lou X, Sun S, Chen W, Zhou Y, Huang Y, Liu X, et al Negative feedback regulation of NF-kappaB action by CITED2 in the nucleus. J Immunol. 2011;186(1):539–48. Epub 2010/11/26. 10.4049/jimmunol.1001650 21098220

[55] Lopes Floro K, Artap ST, Preis JI, Fatkin D, Chapman G, Furtado MB, et al Loss of Cited2 causes congenital heart disease by perturbing left-right patterning of the body axis. Human molecular genetics. 2011;20(6):1097–110. 10.1093/hmg/ddq554 21224256

[56] Xu B, Qu X, Gu S, Doughman YQ, Watanabe M, Dunwoodie SL, et al Cited2 is required for fetal lung maturation. Dev Biol. 2008;317(1):95–105. 10.1016/j.ydbio.2008.02.019 18358466 PMC2467387

[57] Li Q, Hakimi P, Liu X, Yu WM, Ye F, Fujioka H, et al Cited2, a transcriptional modulator protein, regulates metabolism in murine embryonic stem cells. J Biol Chem. 2014;289(1):251–63. 10.1074/jbc.M113.497594 24265312 PMC3879548

[58] Sakai M, Matsumoto M, Tujimura T, Yongheng C, Noguchi T, Inagaki K, et al CITED2 links hormonal signaling to PGC-1alpha acetylation in the regulation of gluconeogenesis. Nat Med. 2012;18(4):612–7. 10.1038/nm.2691 22426420

[59] Li Q, Ramirez-Bergeron DL, Dunwoodie SL, Yang YC. Cited2 gene controls pluripotency and cardiomyocyte differentiation of murine embryonic stem cells through Oct4 gene. J Biol Chem. 2012;287(34):29088–100. 10.1074/jbc.M112.378034 22761414 PMC3436543

[60] Chou YT, Wang H, Chen Y, Danielpour D, Yang YC. Cited2 modulates TGF-beta-mediated upregulation of MMP9. Oncogene. 2006;25(40):5547–60. Epub 2006/04/19. 10.1038/sj.onc.1209552 16619037

[61] Leong DJ, Li YH, Gu XI, Sun L, Zhou Z, Nasser P, et al Physiological loading of joints prevents cartilage degradation through CITED2. FASEB J. 2011;25(1):182–91. 10.1096/fj.10-164277 20826544 PMC3005439

[62] Lee JY, Taub PJ, Wang L, Clark A, Zhu LL, Maharam ER, et al Identification of CITED2 as a negative regulator of fracture healing. Biochem Biophys Res Commun. 2009;387(4):641–5. 10.1016/j.bbrc.2009.07.029 19607804 PMC3008352

[63] Raychaudhuri S. MicroRNAs overexpressed in growth-restricted rat skeletal muscles regulate the glucose transport in cell culture targeting central TGF-beta factor SMAD4. PLoS One. 2012;7(4):e34596 10.1371/journal.pone.0034596 22506032 PMC3323545

