# Supplementary figures and images for: The Signature of MicroRNA Dysregulation in Muscle Paralyzed by Spinal Cord Injury Includes Downregulation of MicroRNAs that Target Myostatin Signaling
Source: PLoS One. 2016 Dec 1;11(12):e0166189. doi: 10.1371/journal.pone.0166189 (PMC5132212; doi:10.1371/journal.pone.0166189)

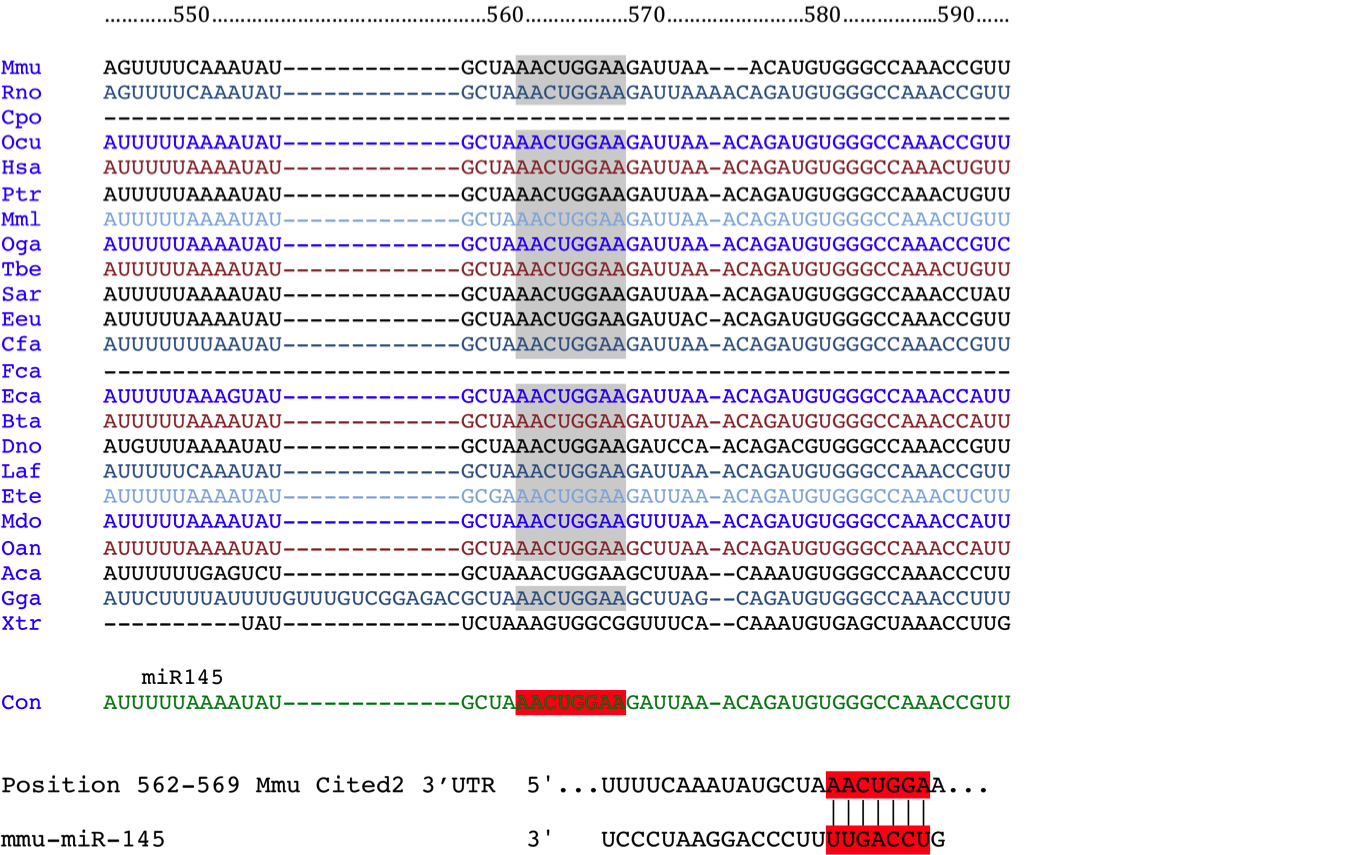

Supplement: S1 Fig — Upper panel shows a sequence alignment of the region of mouse (Mmu) Cited2 mRNA containing the predicted miR-145 seed sequence; the seed sequence is highlighted in light blue. Lower panel: an alignment of murine cited2 mRNA and murine miR-145 is shown. The seed sequence within the Cited2 3’-UTR is highlighted in red. (TIFF) [file pone.0166189.s001.tiff]
